# Childhood Sexual Trauma and Antiretroviral Therapy Adherence: A Mixed-Methods Systematic Review

**DOI:** 10.1007/s10461-020-03009-7

**Published:** 2021-02

**Authors:** Monique J. Brown, Andrea D. Brown, Mohammad Rifat Haider, Amy Edwards, Elizabeth Crouch, Xiaoming Li

**Affiliations:** 1 Department of Epidemiology and Biostatistics, Arnold School of Public Health, University of South Carolina, Columbia, SC, USA; 2 South Carolina SmartState Center for Healthcare Quality, Arnold School of Public Health, University of South Carolina, Columbia, SC, USA; 3 Rural and Minority Health Research Center, Arnold School of Public Health, University of South Carolina, Columbia, SC, USA; 4 Office for the Study on Aging, Arnold School of Public Health, University of South Carolina, Columbia, SC, USA; 5 Department of Social and Public Health, College of Health Sciences and Professions, Ohio University, Athens, OH, USA; 6 Thomas Cooper Library, University of South Carolina, Columbia, SC, USA; 7 Department of Health Services Policy and Management, Arnold School of Public Health, University of South Carolina, Columbia, SC, USA; 8 Department of Health Promotion, Education, and Behavior, Arnold School of Public Health, University of South Carolina, Columbia, SC, USA

**Keywords:** child abuse, sexual assault, violence exposure, sexual abuse, ART adherence

## Abstract

Childhood sexual abuse (CSA) has been shown to be more prevalent among populations living with HIV. Antiretroviral therapy (ART) adherence is crucial for populations living with HIV as it significantly increases the likelihood of attaining and maintaining viral suppression. Previous findings on the association between CSA and ART adherence have been mixed. The current mixed-methods systematic review aimed to identify quantitative and qualitative studies from CINAHL, PsycInfo, PubMed, and Web of Science examining the relationship between CSA and ART adherence. Authors were also contacted if relevant data were unpublished. Studies had to be published from January 1, 2000 to April 1, 2019, written in English, and examined CSA as an exposure and ART adherence as an outcome. Four domains were combined: 1) childhood sexual abuse; 2) child; 3) antiretroviral; and 4) adherence. Eight quantitative and two qualitative studies were retained. The results showed that four quantitative studies found no association while the other four found factors such as timing of victimization, mental health and gender influenced the association between CSA and ART adherence. Themes emerging from the qualitative studies included use of ART evoking memories of CSA; CSA impacting mental health; and mental health treatment improving ART adherence. Mixed insights included the intricate links between CSA and ART adherence and the role of external factors on the relationship. ART adherence intervention programs may be needed for people who have experienced CSA. However, future studies are needed that will examine the association between CSA and ART adherence and include subgroup analyses.

## Resumen

El abuso sexual infantil es más prevalente en personas con VIH. La adherencia a la terapia antirretroviral (TAR) es crucial para personas con VIH porque incrementa la probabilidad de alcanzar y mantener la supresión viral. Resultados previos de la asociación entre el abuso sexual infantil y la adherencia a la TAR han sido variados. El objetivo de esta revisión sistemática utilizando métodos mixtos fue identificar investigaciones cuantitativas y cualitativas en CINAHL, PsycInfo, PubMed y Web of Science que exploran la relación entre el abuso sexual infantil y la adherencia a la TAR. Contactamos a los autores si no se publicaron datos relevantes. Las investigaciones tuvieron que ser publicadas desde enero 1 de 2000 hasta abril 1 de 2019, escritas en inglés, y explorando el abuso sexual infantil como la exposición y la adherencia a la TAR como el resultado. Cuatro ámbitos fueron combinados: 1) el abuso sexual infantil; 2) el niño; 3) el antirretroviral; y 4) la adherencia. Ocho estudios cuantitativos y dos estudios cualitativos fueron retenidos. Los resultados demostraron que cuatro estudios cuantitativos no mostraron asociación mientras los otros cuatros mostraron que los factores como el momento de victimización, la salud mental y el género influyeron en la asociación entre el abuso sexual infantil y la adherencia a la TAR. Los temas emergentes de los estudios cualitativos incluyeron el uso de la TAR que evoca recuerdos del abuso sexual infantil; el abuso sexual infantil tiene un impacto en la salud mental; y el tratamiento de salud mental mejora la adherencia a la TAR. Ideas mixtas incluyeron las relaciones complejas entre el abuso sexual infantil y la adherencia a la TAR, y el papel de los factores externos en la relación. Los programas de intervención para la adherencia a la TAR, tal vez, son necesitados para las personas que tuvieron la experiencia del abuso infantil. Sin embargo, se necesitan investigaciones futuras que examinen la asociación entre el abuso sexual infantil y la adherencia a la TAR incluyendo análisis de subgrupos.

## Introduction

People living with HIV (PLWH) have been shown to be increasingly likely to have a history of childhood sexual abuse (CSA) (Mugavero et al., 2007; Whetten et al., 2006). Previous research estimates show that between 8% to 31% of girls and 3% to 17% of boys have experienced CSA worldwide (Barth, Bermetz, Heim, Trelle, & Tonia, 2013). However, compared to the general population, rates of CSA among PLWH are quite high and are estimated to range from 31 to 53% for women living with HIV (McCall, Lauridsen-Hoegh, Unger, Phillips, & Kille, 2018; Willie, Overstreet, Sullivan, Sikkema, & Hansen, 2016) and from 19% to 70.8% for men living with HIV (Brennan, Hellerstedt, Ross, & Welles, 2007; Kamen, Bergstrom, Koopman, Lee, & Gore-Felton, 2012; Markowitz et al., 2011; Pantalone, Horvath, Hart, Valentine, & Kaysen, 2015; Whetten et al., 2012).

### The syndemic of childhood sexual abuse and other forms of trauma

Populations who experience CSA often undergo multiple forms of trauma in their lifetime, including physical abuse, emotional abuse, and intimate partner violence (Dong, Anda, Dube, Giles, & Felitti, 2003). Increased severity and frequency of experience with CSA and other forms of trauma can influence greater likelihood of poor health outcomes among those affected (Adams, Mrug, & Knight, 2018; Boudewyn & Liem, 1995; Zanarini et al., 2002). Particularly, previous research has found that exposure to adverse events such as CSA is linked to substance abuse, future chronic disease development, and the development of mental health problems such as PTSD and depression (Batchelder et al., 2017; Brown et al., 2017; Brown, Perera, Masho, Mezuk, & Cohen, 2015; Danese et al., 2009; Markowitz et al., 2011; Pretty, O’Leary, Cairney, & Wade, 2013; Spies et al., 2012). Those who have experienced CSA are also more likely to have increased hospitalizations, disease progression and mortality, especially among PLWH (Pence et al., 2012).

### Experiencing childhood sexual abuse and risky behaviors

High rates of CSA among PLWH should be given marked consideration given that past research has shown that this population is likely to engage in risky sexual behaviors (Anaya, Swendeman, & Rotheram-Borus, 2005; Brown, Masho, Perera, Mezuk, & Cohen, 2015; Pence et al., 2012; Whetten et al., 2012). Cohen et al.’s (2000) study using 1,288 women living with HIV and 357 HIV-negative women aged 18 and older found that CSA was associated with several risky sexual behaviors, including having greater than 10 lifetime male sexual partners, having had sexual intercourse with a male or female partner at risk for HIV acquisition, and having had sex for money, drugs, or shelter. (Cohen et al., 2000). Black and White populations living with HIV, and PLWH who abuse substances are known to most frequently report CSA (Anaya et al., 2005; Kidman & Violari, 2018; Markowitz et al., 2011; Paxton, Myers, Hall, & Javanbakht, 2004). Several studies have also found that CSA is linked with increased risk for engaging in unprotected sex, adult sexual revictimization, and having greater number of sex partners among heterosexual men and men who have sex with men (MSM) living with HIV (Markowitz et al., 2011; O’Leary, Purcell, Remien, & Gomez, 2003; Pantalone et al., 2015; Whetten et al., 2012).

### The importance of antiretroviral therapy adherence

Higher likelihood of engagement in risky sexual behaviors, substance abuse, and mental health problems among PLWH are of great concern given their association with increased likelihood of onward HIV transmission (Colfax et al., 2002; Esser, Krotzek, Dirks, Scherbaum, & Schadendorf, 2017; Fox et al., 2009; Sikkema et al., 2010). Achieving and maintaining HIV viral suppression is, thus, particularly imperative for high risk groups such as PLWH who have experienced CSA. Using antiretroviral therapy (ART) and adhering to prescribed ART regimens is crucial in attaining and maintaining viral suppression, to prevent HIV transmission, and to prevent the advancement of HIV infection (Bartlett, 2002; Bunnell et al., 2006). PLWH taking ART are recommended to adhere to prescribed ARTs regimens at least 95% of the time in order to fully benefit from treatment (Bartlett, 2002).

### Childhood sexual abuse and antiretroviral therapy adherence

Past research suggests that gender, experiencing more CSA events or lifetime traumas, age at CSA, and substance abuse are key influences for poor HIV medication adherence among people living with HIV who have experienced CSA (Markowitz et al., 2011; M. Mugavero et al., 2006; Wilson, Sikkema, & Ranby, 2014). CSA may be intrinsically linked to poor HIV medication adherence due to its association with low social support and trauma symptoms like stress and negative coping (Johnson, Heckman, Hansen, Kochman, & Sikkema, 2009; McCall et al., 2018; Meade, Hansen, Kochman, & Sikkema, 2009; Wilson et al., 2014). Anxiety, depression, and PTSD are also common trauma-related consequences of CSA that have been found to be individually associated with poor ART adherence among PLWH (Sauceda, Wiebe, & Simoni, 2016; Willie et al., 2016).

### Study rationale and aims

Previous research has had mixed results on how CSA may be indirectly or directly linked to poor ART adherence across various populations of PLWH (Kidman & Violari, 2018; Liu et al., 2006; Mugavero et al., 2006; Wilson et al., 2014). Kidman and Violari found that childhood adversity was associated with partner violence victimization, which was linked to poor medication adherence to antiretroviral therapy. CSA severity was associated with substance use, which was related to lower levels of adherence. However, a direct link between severity of CSA and poorer ART adherence was not found (Liu et al., 2006). Lifetime traumatic events, including CSA, were linked to poorer ART adherence (Mugavero et al., 2006) and within the context of CSA, gender may play a moderating role in the influence of psychosocial factors on ART adherence (Wilson et al., 2014). Past reviews have largely focused on assessing the relationship between CSA or childhood trauma in relation to outcomes such as HIV risk (Lloyd & Operario, 2012; McGeough & Sterzing, 2018; Thornton & Veenema, 2015). Therefore, collectively examining research findings on the relationship between CSA and HIV medication adherence is warranted. This paper outlines results from a systematic review on the association between CSA and HIV medication adherence.

## Methods

### Literature search

The Preferred Reporting Items for Systematic Reviews and Meta-Analyses (PRISMA) guidelines (Oxford., 2015) were used to guide this systematic review. Quantitative and qualitative studies that provided information on the relationship between childhood sexual trauma and ART adherence were retrieved. The published peer-reviewed literature from January 1, 2000 to April 1, 2019 was searched in four databases: CINAHL, PsycInfo, PubMed, and Web of Science. Search algorithms were developed with assistance from a public health librarian (AE) and combined four domains: 1) childhood sexual abuse; 2) child; 3) antiretroviral; and 4) adherence. The search was expanded to include specific text words. The search algorithm included only English publications. Study selection and extraction of data were completed by at least two team members (MJB, ADB, MRH, AE).

### Review Process

Four reviewers participated in the first and second phases of review (MJB, ADB, MRH, AE). If there was a disagreement between two reviewers in each phase, a third reviewer made the final decision.

#### First phase of review:

Four reviewers reviewed the titles and abstracts of articles independently. All studies were retained for further review if the title and/or abstract included concepts related to “HIV”, “trauma”, “abuse” or “adherence”.

#### Second phase of review:

The full texts for all review studies and primary studies that were considered relevant were retrieved. If additional data were needed that were not found in articles, the investigators were contacted to obtain these data. This method of obtaining data had a 57.1% success rate (4/7).

**Figure 1.**
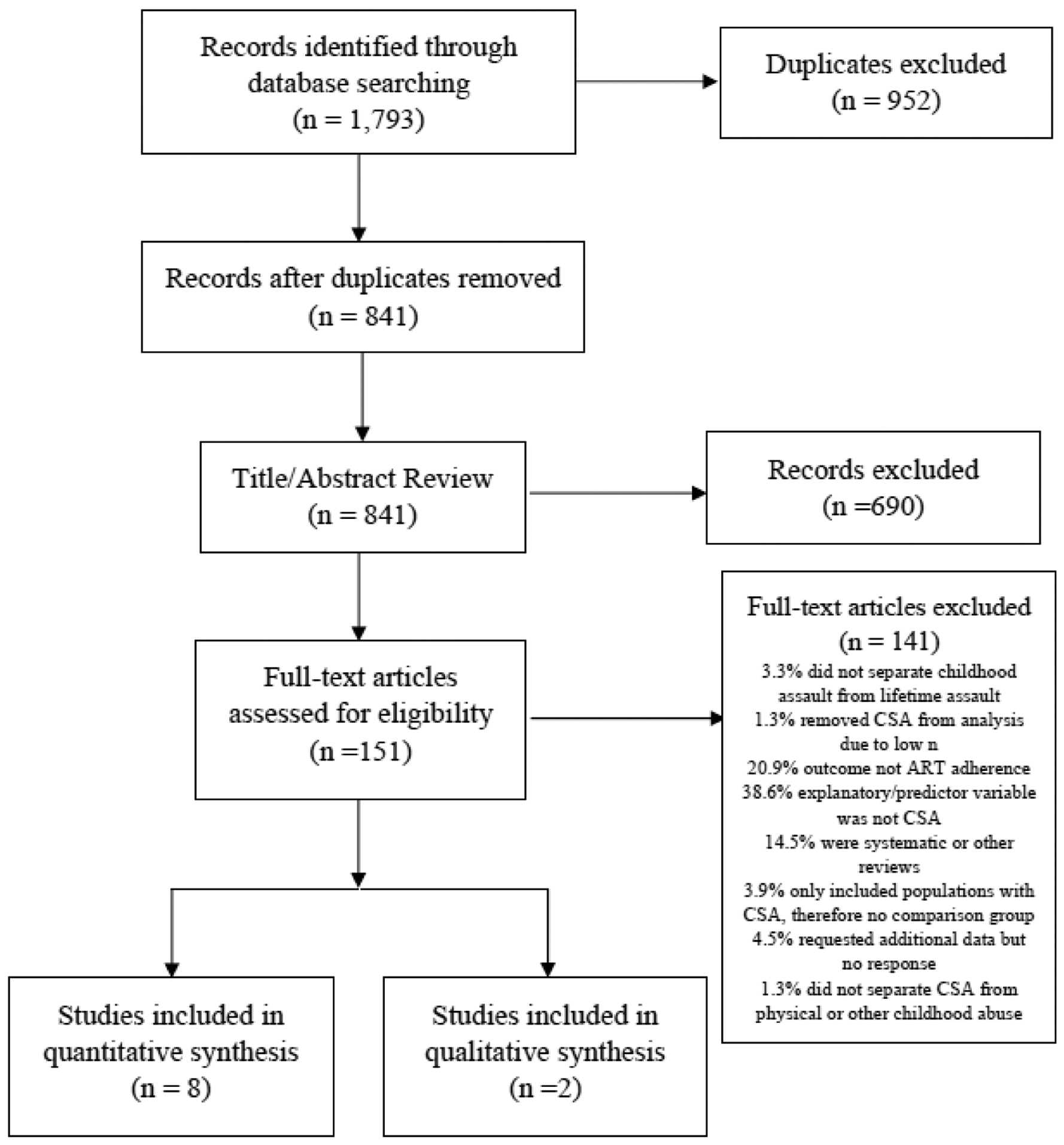
Flow diagram for article selection for systematic review

As shown in Figure 1, a total of 1,793 records were identified and after duplicates were removed (n=952), the titles and abstracts of 841 articles were screened. A total of 690 were excluded after title/abstract review. Therefore, the full text of 151 articles were assessed. There were 141 studies excluded, which resulted in eight (8) articles to be included in a quantitative synthesis, and two (2) for qualitative synthesis. The research team captured relevant information from the studies to be included in final tables.

### Descriptive synthesis

The research team developed shell tables to include pertinent information about the studies. The PRISMA guidelines as well as the research questions were used as a guide to determine what information to include in the tables. For the quantitative studies (Table 1), information garnered included: definition of CSA, study purpose/questions, geographic location, source of sample, number of participants in the sample, methods, and main findings. For the qualitative studies (Table 2), information obtained included: research theoretical framework, study purpose/questions, geographic location, source of sample, methods and main findings. Shared or mixed insights were also obtained from quantitative and qualitative studies. Qualitative data were analyzed with a framework thematic analysis (Kimera et al., 2019).

### Quality assurance of systematic review

At least two reviewers were responsible for evaluating the titles, abstracts and full texts of relevant articles. Research team meetings and group discussions were held throughout the extraction process to raise and discuss any related issues, which came up.

## Results

### Types of participants

Participants included people living with HIV who were on ART. Study participants were obtained from a variety of settings: a sample of PLWH enrolled in a primary care-based alcohol intervention study in San Francisco; women from the Canadian HIV Women’s Sexual Health and Reproductive Health Cohort Study and the Women’s Interagency HIV Study (WIHS) in the US; PLWH from the Coping with HIV/AIDS In Tanzania (CHAT) longitudinal cohort study; PLWH from the Centro de Salud Familiar La Fe Care Center, a federally qualified health center in El Paso Texas; urban university-affiliated outpatient clinics in the Northwestern US; participants from substance use treatment centers in Massachusetts and Rhode Island; and from street outreach in East Harlem, New York.

### Quantitative findings

A total of eight quantitative studies were found to meet inclusion criteria for this review. Although this review assessed studies from 2000 to 2019, the final eight quantitative studies that were eligible for inclusion were published between 2008 to 2018. Results for the eight quantitative studies can be seen in Table 1. The samples varied greatly.

Across study samples, reported CSA among participants ranged from 6.8% to 55%. No two studies used the same method to measure CSA. For HIV medication adherence, six studies used self-reported measures of adherence either over the past week, month, three months, or six months.

Findings on associations between CSA and HIV medication adherence varied across studies. Particularly, Whetten’s 2013 study among 451 men and women living with HIV in Tanzania found that compared to participants who had not experienced sexual abuse before puberty, participants who had experienced sexual abuse before puberty were 78% more likely to report incomplete adherence or non-adherence to ART (RR = 1.78, 95% CI: 1.13, 2.79, p = .0120). The Markowitz et al. (2011) study among 119 men and women living with HIV with history of drug abuse found that experience of CSA both before the age of 13 *and* between age 13 to 16 years old was associated with poor HIV medication adherence after adjustment for confounders (β = −0.20, *p* = 0.04). However, experiences of CSA either before the age of 13 *or* between age 13 to 16 years old was not linked to poor HIV medication adherence. Kang’s 2008 study using 220 men and women living with HIV who were drug users found that CSA was associated with decreased self-reported use of HIV medications after adjustment for confounders (OR=0.51; 95%CI: 0.27,0.96, *p*=0.04). Yet after stratifying by gender, only men were found to have a significant association between CSA and use of HIV medications (OR=0.49, *p* = 0.04). Preliminary analysis from Carter’s large cohort study found that women who reported CSA had slightly lower unadjusted odds of optimal HIV medication adherence, but this result was not statistically significant (OR=0.933; 95% CI: 0.705–1.236, *p* = 0.630). Likewise, Young-Wolff’s 2019 study among 566 men and women living with HIV who were at risk for alcohol abuse found no statistically significant association between CSA and HIV medication adherence (adjusted OR=0.95 ;95% CI: 0.52, 1.71, *p* = 0.855). Pantalone’s 2015 study also found that among 166 MSM living with HIV, there was no statistically significant association between CSA and HIV medication adherence (adjusted OR= 1.11; 95% CI: 0.48, 2.56, *p* = 0.814).

Dale’s 2014 study among a sample of 139 women living with HIV used a composite measure of any history of abuse, which included CSA, adult sexual abuse, physical abuse, or domestic violence. Overall, this study found that there was no statistically significant association between any history of abuse and HIV medication adherence. However, when considering interaction between resilience and history of sexual abuse in childhood and/or adulthood, increase in resilience among women with history of sexual abuse in childhood and/or adulthood was associated with an increase in ≥95% HIV medication adherence (OR=1.15; 95% CI 1.02–1.29). Similarly, Sauceda’s 2016 study using Latino MSM living with HIV found that depression significantly mediated the relationship between CSA and poor HIV medication adherence, yet worse adherence was found in CSA participants with the lowest resilience percentile indexes. Mean difference on HIV medication adherence between CSA groups through the indirect effect of depression equaled to −6.77 (SE=3.44), −4.48 (SE=2.36) and −2.96 (SE=1.71) at the 10th, 25th, and 50th percentile of the resilience index, respectively.

### Qualitative findings

Overall, only two qualitative studies met inclusion criteria for this review. One study included eight women living in Cape Town, South Africa. This study aimed to examine the lives of women who had experienced any history of sexual abuse. A key theme discovered through this study were that women felt that using HIV ART evoked memories of their experience with CSA. The other study included three participants from psychiatric care at an HIV clinic. This case study aimed to investigate the relationship between childhood trauma, PTSD, and medication adherence. Study results showed that all participants who experienced sexual abuse additionally suffered from depression and PTSD. Psychodynamic therapy sessions for the male participant led to HIV medication adherence. One of the two female participants did not adhere to HIV medications.

### Integration

**Figure 2.**
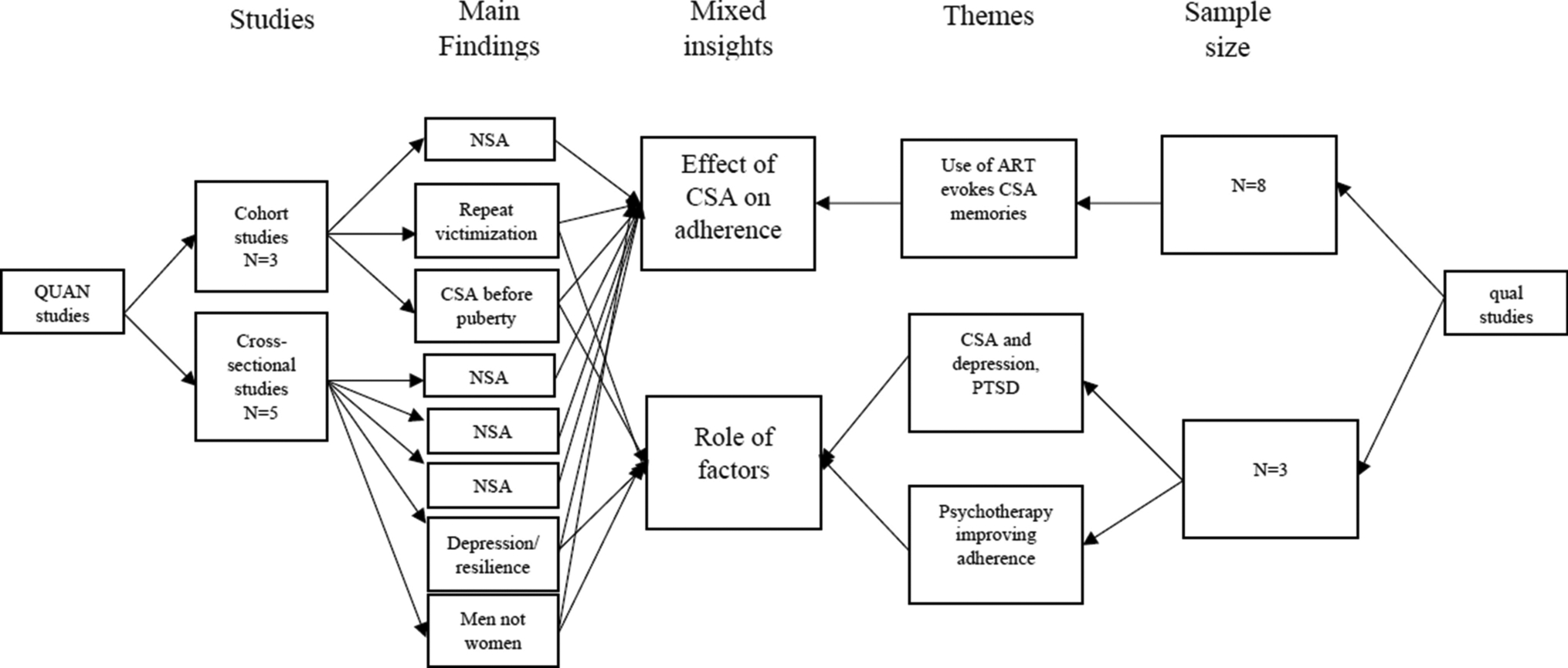
Mixed-methods systematic review, analysis and integration. NSA: No statistically significant association

Data from both quantitative and qualitative studies were used to obtain mixed insights. Based on the themes from the qualitative studies and analyses in quantitative studies, mixed insights included: 1) the intricate link of the effect of CSA on taking ART as prescribed; and 2) the role that external factors such as mental health, mental health treatment, substance use, and repeat victimization may play in the relationship between CSA and adherence (Figure 2).

## Discussion

To our knowledge, this is the first study to perform a systematic review on the association between CSA and ART adherence. Previous reviews examined the association between CSA or childhood trauma in relation to outcomes such as HIV risk. Overall, we found that data allowing the estimation of the association between CSA and ART adherence were sparse. This lack of research could be due to the sensitive nature of CSA and challenges in accurately measuring ART adherence. We propose that more studies address this relationship acknowledging the sensitive nature of CSA and relying less on self-report of ART adherence with the use of biomarkers.

The majority of quantitative studies included were cross-sectional studies and only three were cohort studies. Only two qualitative studies were found with very small sample sizes (N=8 and N=3). Therefore, this systematic review was most akin to the mixed-methods explanatory sequential design with an emphasis on the quantitative data (Figure 2). For a summary of the critical findings and implications, see Table 3 and Table 4, respectively.

Although majority of the cross-sectional studies found no statistically significant association between CSA and ART adherence, one cross-sectional study examining sex as a moderator of the relationship between CSA and ART adherence found that the relationship was statistically significant among men but not among women (Kang et al., 2008). This study suggests that sex differences may be present when examining the association between CSA and ART adherence and should be considered as an important factor in future studies.

Additional important factors that became evident, especially from the integrated analysis, was the potential role that external factors played in the relationship between CSA and ART adherence: mental health, mental health treatment, substance use history, repeat victimization, and CSA occurring before puberty. For example, the qualitative studies found that depression and PTSD were important factors to consider in this relationship. Furthermore, women found that taking their ART medication resulted in memories of their experience with CSA. Therefore, future ART adherence interventions may need to consider improving coping mechanisms, especially for women who were exposed to CSA. Finally, an additional interesting finding was that repeat sexual abuse before age 13 and between ages 13 and 16 was found to be predictive of poorer medication adherence among women and men living with HIV. Another study, however, found that CSA before puberty was also associated with lower ART adherence. These findings suggest that repeat sexual abuse revictimization and timing of CSA may play an important role in lowering ART adherence and should also be a focal point in ART adherence interventions.

### Limitations

There are some limitations to consider when interpreting the study’s findings. Only eight quantitative and two qualitative studies were included in the study. The research team also endeavored to contact investigators with a 57% success rate with 43% not responding to requests or indicating that there was no time available to conduct additional analyses. Nevertheless, this success rate was better than previous research with a response rate of 25% (Kimmes et al., 2019).

### Methodological Limitations.

We were unable to conduct a meta-analysis due to the diversity and stark differences in the studies, especially in the operationalization of key variables such as CSA and ART adherence. For example, CSA operationalizations ranged from using sexual-abuse related items on an ACE questionnaire of CSA experiences before age 18 to reports of experiencing sexual abuse at age 12 or younger. Based on our findings, we suggest operationalizing CSA based on related experiences before age 18: someone at least 5 years older touching the respondent sexually or forcing the respondent to touch them sexually; and/or being forced to have sex (including oral sex, anal or vaginal sexual intercourse). Diversity was also seen in the operationalization of ART adherence with operationalizations ranging from self-report of ART adherence in the past month using the visual analog scale to self-report of ART adherence in the past week. The percentage of optimal adherence also varied from >90% to >95%. These findings suggest that standardized measures of CSA and ART adherence are needed and when available should be used in order to facilitate better comparisons across studies. Indeed, Mathews and Collin-Vézina (2019) proposed a conceptual model of CSA to distinguish CSA from other concepts. One quantitative study was also conducted in Canada. Comparisons across countries due to cultural differences, if done, should be done with caution (Winzer, Krahé, & Guest, 2019). Diversity was also present in the samples of the studies with regards to age, gender, sexual orientation, and race/ethnicity.

## Conclusions

The small number of relevant studies indicate that more research examining the impact of sexual trauma on ART adherence is needed. For example, there are no studies examining the impact of CSA on HIV treatment outcomes such as ART adherence or viral suppression among older adults. Filling this gap of the literature is crucial as people are living longer with HIV and CSA is a common traumatic event among people living with HIV. Kimera et al. suggests that additional studies examining the impact of CSA on health behaviors are needed so as to develop the necessary interventions to improve healthy behaviors and medication adherence among youths living with HIV (Kimera et al., 2019). Whetten et al. states that additional research into trauma and its related risk factors and consequences is warranted, especially with respect to HIV risk behaviors and ART adherence (Whetten, Whetten, Ostermann, & Itemba, 2008). The Living in the Face of Trauma (LIFT) intervention by Sikkema and colleagues (2013), which was found to reduce unprotected sexual intercourse among participants living with HIV with CSA histories, may provide some intervention components for future consideration to improve ART adherence. These may include: using a group-based approach, helping participants to identify and address stressors related to living with HIV and their trauma history; and taking part in related discussions and skill building exercises (Conduent, 2020). We also recommend future studies on CSA and ART adherence conduct subgroup analyses, for example, by gender and mental health status. In addition, due to the lack of studies among older adults and that this population continues to grow, we argue that these studies are also warranted for adults aging with HIV/AIDS.

## Supplementary Material

10461_2020_3009_MOESM1_ESM

## Figures and Tables

**Table 1.** Quantitative studies examining the relationship between childhood sexual trauma and antiretroviral therapy adherence

References	Definition of CSA	Study purpose/ques tions	Geographic location	Source of sample	Sample	Methods	Main Findings
1. Young-Wolff (2019)	Measured using questions on a 10-item ACE questionnaire adapted from the original ACE study (“Got your ACE score”, 2018). Items on questionnaire measure experiences of sexual abuse and other ACEs that occurred before the age of 18.	Examined the association between adverse childhood experiences (ACEs), depression and anxiety symptoms, substance use, antiretroviral therapy (ART) adherence, and HIV-clinical indicators.	San Francisco, CA	Used data from “The Health and Motivation Study” randomize d control trial. Participant s were all enrolled in a primary care-based alcohol interventio n study.	584 HIV positive men and women who were at risk for unhealth y alcohol use (sample 96.9% male, and 63.0 non-Hi spanic white)	Cross-sectional study Data collected through electronic health record, baseline and 12-month follow-up interviews HIV medication adherence was self-reported and Dichotomize d to < or ≥90%adhere nee	Overall, 12.9% of participant s reported ART adherence <90% (18 people living with HIV (PLWH) missing adherence data) Out of 566 total participant s, 25% of participant s reported CSA No significant association was found between CSA and ART adherence before (OR=0.95 (95% CL (0.54, 1.68), p = 0.869) and after adjustment for confounder s (OR=0.95 (95% CI: (0.52, 1.71), p = 0.855)
2. Carter (2018)	Measured through question asking “any experience of physical, sexual, verbal, or controlling violence in the past 3 months” (yes vs. no).	Used the substance abuse, violence and HIV/AIDS (SAVA) theoretical framework in combination with Latent Class Analysis (LCA) methodology to model multiple types of substance use simultaneousl y and examine associations with sub-optimal HIV cART adherence, interpersonal violence, and other social determinants of health.	British Columbia, Ontario, or Quebec, Canada	The Canadian HIV Women’s Sexual and Reproductive Health Cohort Study (CHIWOS). Recruited sample through peers, HIV clinics, AIDS Service Organizati ons, online networks, and other methods.	N=1,363 HIV positive women age 16 or older	Cohort Study Participants recruited Between August 27, 2013 and May 1, 2015 Peer Research Associates (i.e. HIV positive women with research training), administered questionnaires to Participants in English or French using FluidSurvey sTM software. Baseline visit took place either in-person at clinic community sites, or women’s homes, or via phone/Skype. Initial visits lasted a median of 120 min (interquartile range (IQR): 90–150). Wave 2 (18- month) follow-up interviews were completed in February 2017, and wave 3 (36- month) visits were currently inprogress. HIV medication adherence in the past month before visit measured via self-report.	Among 1,064 women living with HIV who responded to this question, 425 (39.9%) reported childhood sexual violence. Sixty-one percent (61%) reported being ≥95% adherent to ART. Women who reported childhood sexual violence had Slightly lower unadjusted odds of optimal ART adherence, although the confidence interval includes (1 OR=0.933 ; 95% Cl: 0.705–1.236; p-value=0.630).
3. Sauceda (2016)	Based on Sexual experiences prior to the age of 16 years, participants reported a history of: 1) no sex, 2) consensual sex, or 3) forced sex.	While Considering resilience as a moderator, tested depression as a mediator between the association of CSA and HIV medication adherence.	El Paso, Texas	Recruited from the Centro de Salud Familiar La Fe CARE Center, a federally qualified health center that offers comprehen sive HIV and AIDS services to predominately Mexican-American individuals living on the U.S.-Mexico Border.	N= 149 HIV positive, Latino men who have sex with men (MSM), who were biologically bom male and age 18 or older	Cross-Sectional study Data collected through in-person interviews that were conducted in either English or Spanish from 2012 to 2013. Self-reported past 30-day adherence to ART measured by the visual analog scale (VAS).	In total, 34% of participant s who were sexually active before age 16 (n=99) reported at least one non-consensual sexual experience Depression significantl y mediated The relationshi p between CSA and ART adherence, with worse adherence for participant s with the lowest resilience percentile indexes (e.g. mean difference on adherence between CSA groups through the indirect effect of depression equaled to −6.77 (SE=3.44), −4.48 (SE=2.36) and −2.96 (SE=1.71) at the 10^th^’ 25^th^, and 50^th^ percentile of the resilience index).
4. Pantalone (2015)	Used two items from the sexual abuse subscale of the Childhood Maltreatment Interview Schedule—Short Form (CMIS-SF; Briere, 1992) to assess behaviorally-defined penetrative or nonpenetrative CSA (i.e., sexual abuse before the age of 18 by someone who was 5+ years older and/or by someone of any age who used force or coercion).	Investigated The relationship between syndemic indicators (including mental health, physical abuse, CSA, and substance abuse), and key HIV health outcomes such as HIV medication nonadherence, sexual risk behaviors (e. g. condomless anal sex), and healthcare utilization.	Northwest, United States	Participant s were recruited from two urban, public, university-affiliated outpatient HIV clinics.	N= 166 HIV positive, biologically bom male MSM, age 18 and older.	Cross-Sectional study Collected survey data via computer-assisted self interview (CASI). HIV medication adherence measured by self-reported number of doses missed in the week prior to assessment.	Overall, 38.6% of participant s reported behavioral indicators of CSA. No significant association was found between CSA and HIV medication adherence before (OR= 1.53, (95% Cl: 0.71, 3.33), p = 0.281) and after adjustment for confounder s (adjusted OR= 1.11, (95% Cl: 0.48, 2.56), p = 0.814).
5. Dale (2014)	Women were asked at baseline questions to capture any history of prior adult and/or childhood (before age 18) sexual abuse, (0=no reported history, 1 =reported history of abuse), and current versus previous occurrence of sexual abuse (l=current abuse, 0=no current abuse).	Analyze how resilience interacts with abuse history in relation to HIV medication adherence, HIV viral load (VL), and CD4+ cell count.	Boston, MA	The Ruth M. Rothstein CORE Center/Coo k County Health and Hospital Systems site of the Women’s Interageney HIV Study (WIHS)), a longitudina 1 study of women with and at risk for HIV.	N= 139 HIV positive women age 16 and older	Cross-sectional sub study Data collected from questionnaires that women completed during one regular 6 month visit from 2011 to 2012. Women were asked how often they took their highly active antiretroviral therapy (HAART) medication as prescribed during the 6 months before the current study visit.	Of the 138 women, 55% percent reported a history of adult and/or childhood sexual abuse. No significant association s were found between current abuse or any histories of abuse (sexual abuse, physical abuse, or domestic violence) with HAART adherence. Among women with a history of sexual abuse, each unit increase in Resilience was associated with 1.15 increase in the odds of ≥95% HAART adherence (95% CI: 1.02,1.29).
6. Whetten (2013)	The Life Events Checklist was used to measure sexual abuse. Sexual abuse is defined in the scales to include sexual experiences (e.g., touching, intercourse) where force or threat of force was used; additionally, in children before the age of puberty the threat of force or harm was implied by a 5-year age difference between victim and perpetrator	To predict Factors associated with incomplete self-reported adherence to ART among people living with HIV in Tanzania.	Kilimanjar o, Tanzania	The Coping with HIV/AIDS in Tanzania (CHAT) longitudina 1 cohort study in the Kilimanjar o Region. Participants were recruited from two local hospital-based HIV clinics and four free standing voluntary HIV Counselling and testing sites.	N= 451 men and women living with HIV, aged 18 and older	Cohort study Used data collected at 36-month follow-up. Baseline data collected in 2008 to 2009 ART adherence over the past 3 months was assessed with three self-report questions: 1) When was the last time that they had missed a dose of their antiretroviral s; 2) The participant was asked to show on a 0–100% visual analog scale the percentage of their antiretroviral drugs they had taken in the last month and 3) the percentage of their antiretroviral drugs they had missed in the last month.	Found that 6.8% of participant s who completely adhered to ART and 17.5% of participant s who did not adhere to ART had experience d sexual abuse before puberty (statistically significant difference, p=.0020). Compared to participant s who had not experience d sexual Abuse before puberty, participants who had experienced sexual abuse before puberty were 78% more likely to report incomplete adherence or non-adherence to ART (RR = 95% Cl: 1.13, p = .0120)
7.Markowitz (2011)	Participants were asked two questions about CSA: (1) When you were 12 years old or younger, did you have a sexual experience with a person who was at least 5 years older than you? and (2) When you were between the ages of 13 and 16, did you ever have a sexual experience with someone who was 10 years older or more?	Examine the association of CSA with (1) sexual risk-taking with an HIV-uninfected or HIV status unknown partner in the past 6 months, (2) heroin use in the last 30 days, and (3) HIV medication adherence.	Massachus etts and Rhode Island	Participants were recruited from substance abuse treatment centers, HIV clinics, and through newspaper ads from 2005 to 2008. The last follow up occurred in 2009.	N= 119 men and women living with HIV with a history of injection drug abuse or depende nee and who were aged 18–65	Mixed cohort study Participants completed a baseline diagnostic assessment during enrollment for a randomized control trial of cognitive behavioral therapy for treating depression and HIV medication adherence but had not yet been randomized. Screening data included questions on CSA. A medication event monitoring system (MEMS) was used to measure the weekly percentage of HIV medication taken on time.	Sexual abuse at or before age 12 was reported by 27. l% of women and 31% of men, whereas sexual abuse between the ages of 13 and 16 was reported by 41.7% of women and 40.8% of men. Sexual abuse during both age ranges was reported by 22.9% of women and 23.9% of men. There were no statistically significant differences in the history of CSA ‘ between women and men. After controlling for gender, Caucasian participant s had a significantl y lower likelihood of a self-reported history of CSA (OR = 0.48, p = 0.048, 95% Cl = 0.23–0.99) Self-report of CSA both before age 12 and between ages 13 and 16 was predictive of poorer medication adherence (β = −0.20; p = 0.04). Self-report of CSA both before age 12 and between ages 13 and 16 did not individuall y predict poorer medication adherence.
8. Kang (2008)	Eleven sexual and physical abuse items from the Childhood Trauma Questionnair e (CTQ: Bernstein et al., 1994). were selected. A dichotomized childhood sexual abuse variable (never/somet imes versus often) was used in analyses.	Examine: (1) differences in health status and healthcare utilization (including HIV care) between drug-using men and women living with HIV; and (2) factors related to receipt of HIV care.	East Harlem, NY	Streetoutreach	N= 220 African-America n or Hispanic populations living with HIV, who are recent heroin and/or crack cocaine drug users, aged 18 or older.	Data were collected from October 2002 and November 2003 using baseline questionnaires for an intervention study designed to enhance treatment engagement for populations living with HIV and using drugs. Used self-reported use of any HIV medications.	Women were more likely to report childhood sexual abuse (51 versus 39%; p=0.08). For both men and women, childhood sexual abuse was negatively associated with use of HIV medication s (Men: OR=0.49, p=0.04; Women: OR=0.43, p=0.09) Using gender in the model, childhood sexual abuse was negatively related to use of HIV medications (aOR=0.51 , p=0.04 (CI:0.27,0.96))

**Table 2.** Qualitative studies examining the relationship between childhood sexual trauma and antiretroviral therapy adherence

References	Research theoretical framework	Study purpose/questions	Geographic location	Source of sample	Sample	Methods	Main Findings
1. Watt (2017)	None stated	Documenting the experiences of women living with HIV with histories of sexual trauma	CapeTown, South Africa	Clinic patients enrolled in HIV care	N=8 women exposed to sexual trauma	Textual data were analyzed in three steps: Individual memos were summarized and content was organized into main themes Memos were reviewed to identify key themes related to the relationshi p between sexual trauma and HIV care engagement Analytic memos were written for each of the themes using a constant comparativ e method to identify similarities and differences across participants.	**Theme:** Impact of sexual trauma on HIV care engagement Sexual trauma impacted HIV care engagement over time as these participants stated that taking ART brought up memories of their childhood sexual trauma history.
2. Cohen (2001)	None stated	Examine the association between early childhood trauma, PTSD, and different levels of adherence to medical treatment	Not stated	On-site psychiatric care in an ambulatory HIV/AID S clinic	N=3 2 Women and 1 man	Case study	All patients who were sexually abused also had depression and posttraumatic stress disorder. One female patient did not adhere to treatment. One male patient was fully adherent after beginning psychodynamic therapy.

**Table 3.** Critical Findings

Having a CSA history may be associated with lower ART adherence.
The relationship between CSA and ART adherence may be influenced by factors such as gender, mental health, substance use, and mental health treatment.
Taking ART may be linked to memories of CSA, which may lower adherence to ART.
More studies (quantitative and qualitative) examining the relationship between CSA and ART adherence, including subgroup analyses, are needed.

**Table 4.** Implications

ART adherence intervention programs may be needed for people who have experienced CSA.
Targeted ART adherence interventions addressing CSA should consider gender differences, and presence of mental health and substance use disorders, and treatment.
ART adherence for people who have experienced CSA may need to address coping skills and strategies specific to CSA experience. Quantitative and qualitative studies and related subgroup analyses will help to determine the intricate links of the relationship between CSA and ART adherence and how this relationship may differ by specific groups. This approach will help to determine specific target groups for interventions.

## References

[R1] Adams J, Mrug S, & Knight DC (2018). Characteristics of child physical and sexual abuse as predictors of psychopathology. Child Abuse & Neglect, 86, 167–177. doi:10.1016/j.chiabu.2018.09.019 30308347 PMC6289670

[R2] Anaya HD, Swendeman D, & Rotheram-Borus MJ (2005). Differences among sexually abused and nonabused youth living with HIV. Journal of Interpersonal Violence, 20(12), 1547–1559. doi: 1177/0886260505280340 16246916 10.1177/0886260505280340

[R3] Barth J, Bermetz L, Heim E, Trelle S, & Tonia T (2013). The current prevalence of child sexual abuse worldwide: A systematic review and meta-analysis. International Journal of Public Health, 58(3), 469–483. doi:10.1007/s00038-012-0426-1 23178922

[R4] Bartlett JA (2002). Addressing the challenges of adherence. Journal of Acquired Immune Deficiency Syndromes, 29 Suppl 1, S2–10. doi: 10.1097/00126334-200202011-00002 11832696

[R5] Batchelder AW, Ehlinger PP, Boroughs MS, Shipherd JC, Safren SA, Ironson GH, & O’Cleirigh C (2017). Psychological and behavioral moderators of the relationship between trauma severity and HIV transmission risk behavior among MSM with a history of childhood sexual abuse. Journal of Behavioral Medicine, 40(5), 794–802. doi:10.1007/s10865-017-9848-9 28396969 PMC5901662

[R6] Bernstein DP, Fink L, Handelsman L, Foote J, Lovejoy M, Wenzel K,…, Ruggiero J (1994). Initial reliability and validity of a new retrospective measure of child abuse and neglect. American Journal of Psychiatry, 151(8), 11321136. 10.1176/ajp.151.8.1132 8037246

[R7] Boudewyn AC, & Liem JH (1995). Childhood sexual abuse as a precursor to depression and self-destructive behavior in adulthood. Journal of Traumatic Stress, 8(3), 445–459. doi: 10.1007/bf02102969 7582609

[R8] Brennan DJ, Hellerstedt WL, Ross MW, & Welles SL (2007). History of childhood sexual abuse and HIV risk behaviors in homosexual and bisexual men. American Journal of Public Health, 97(6), 1107–1112. doi:10.2105/AJPH.2005.071423 17463386 PMC1874190

[R9] Brown MJ, Masho SW, Perera RA, Mezuk B, & Cohen SA (2015). Sex and sexual orientation disparities in adverse childhood experiences and early age at sexual debut in the United States: Results from a nationally representative sample. Child Abuse & Neglect, 46, 89–102. doi:10.1016/j.chiabu.2015.02.019 25804435 PMC4527947

[R10] Brown MJ, Masho SW, Perera RA, Mezuk B, Pugsley RA, & Cohen SA (2017). Sex disparities in adverse childhood experiences and HIV/STIs: Mediation of psychopathology and sexual behaviors. AIDS and Behavior, 21(6), 1550–1566. doi:10.1007/s10461-016-1553-0 27688144 PMC5896316

[R11] Brown MJ, Perera RA, Masho SW, Mezuk B, & Cohen SA (2015). Adverse childhood experiences and intimate partner aggression in the US: sex differences and similarities in psychosocial mediation. Social Science and Medicine, 131, 48–57. doi:10.1016/j.socscimed.2015.02.044 25753285 PMC4479130

[R12] Bunnell R, Ekwaru JP, Solberg P, Wamai N, Bikaako-Kajura W, Were W, … Mermin J (2006). Changes in sexual behavior and risk of HIV transmission after antiretroviral therapy and prevention interventions in rural Uganda. AIDS, 20(1), 85–92. doi:10.1097/01.aids.0000196566.40702.28 16327323

[R13] *Carter A, Roth EA, Ding E, Milloy MJ, Kestler M, Jabbari S,…Kaida A (2018). Substance use, violence, and antiretroviral adherence: A latent class analysis of women living with HIV in Canada. AIDS and Behavior, 22(3):971–985. doi: 10.1007/s10461-017-1863-x 28733919

[R14] *Cohen MA, Alfonso CA, Hoffman RG, Milau V, & Carrera G (2001). The impact of PTSD on treatment adherence in persons with HIV infection. General Hospital Psychiatry, 23(5), 294–296. doi: 10.1016/s0163-8343(01)00152-9 11600172

[R15] Cohen M, Deamant C, Barkan S, Richardson J, Young M, Holman S, … Melnick S (2000). Domestic violence and childhood sexual abuse in HIV-infected women and women at risk for HIV. American Journal of Public Health, 90(4), 560–565. doi:10.2105/ajph.90.4.560 10754970 PMC1446192

[R16] Colfax GN, Buchbinder SP, Cornelisse PG, Vittinghoff E, Mayer K, & Celum C (2002). Sexual risk behaviors and implications for secondary HIV transmission during and after HIV seroconversion. AIDS, 16(11), 1529–1535. doi:10.1097/00002030-200207260-00010 12131191

[R17] Conduent. (2020). Living in the Face of Trauma (LIFT). Retrieved from https://cdc.thehcn.net/promisepractice/index/view?pid=3536

[R18] *Dale S, Cohen M, Weber K, Cruise R, Kelso G, & Brody L (2014). Abuse and resilience in relation to HAART medication adherence and HIV viral load among women with HIV in the United States. AIDS Patient Care and STDs, 28(3), 136–143. doi: 10.1089/apc.2013.0329 24568654 PMC3948478

[R19] Danese A, Moffitt TE, Harrington H, Milne BJ, Polanczyk G, Pariante CM, … Caspi A (2009). Adverse childhood experiences and adult risk factors for age-related disease: Depression, inflammation, and clustering of metabolic risk markers. Archives of Pediatrics and Adolescent Medicine, 163(12), 1135–1143. doi:10.1001/archpediatrics.2009.214 19996051 PMC3560401

[R20] Dong M, Anda RF, Dube SR, Giles WH, & Felitti VJ (2003). The relationship of exposure to childhood sexual abuse to other forms of abuse, neglect, and household dysfunction during childhood. Child Abuse & Neglect, 27(6), 625–639. 12818611 10.1016/s0145-2134(03)00105-4

[R21] Esser S, Krotzek J, Dirks H, Scherbaum N, & Schadendorf D (2017). Sexual risk behavior, sexually transmitted infections, and HIV transmission risks in HIV-positive men who have sex with men (MSM) - approaches for medical prevention. Journal der Deutschen Dermatologischen Gesellschaft, 15(4), 421–428. doi:10.1111/ddg.13217 28294529

[R22] Fox J, White PJ, Macdonald N, Weber J, McClure M, Fidler S, & Ward H (2009). Reductions in HIV transmission risk behaviour following diagnosis of primary HIV infection: a cohort of high-risk men who have sex with men. HIV Medicine, 10(7), 432–438. doi:10.1111/j.1468-1293.2009.00708.x 19459996

[R23] Johnson CJ, Heckman TG, Hansen NB, Kochman A, & Sikkema KJ (2009). Adherence to antiretroviral medication in older adults living with HIV/AIDS: A comparison of alternative models. AIDS Care, 21(5), 541–551. doi:10.1080/09540120802385611 19444661 PMC2736552

[R24] Kamen C, Bergstrom J, Koopman C, Lee S, & Gore-Felton C (2012). Relationships among childhood trauma, posttraumatic stress disorder, and dissociation in men living with HIV/AIDS. Journal of Trauma and Dissociation, 13(1), 102–114. doi:10.1080/15299732.2011.608629 22211444 PMC3298730

[R25] *Kang SY, Goldstein MF, & Deren S (2008). Gender differences in health status and care among HIV-infected minority drug users. AIDS Care, 20(9), 1146–1151. 18608064 10.1080/09540120701842746

[R26] Kidman R, & Violari A (2018). Dating violence against HIV-infected youth in South Africa: Associations with sexual risk behavior, medication adherence, and mental health. Journal of Acquired Immune Deficiency Syndromes, 77(1), 64–71. doi:10.1097/qai.0000000000001569 29040165 PMC5720896

[R27] Kimera E, Vindevogel S, De Maeyer J, Reynaert D, Engelen AM, Nuwaha F, … Bilsen J (2019). Challenges and support for quality of life of youths living with HIV/AIDS in schools and larger community in East Africa: A systematic review. Systematic Reviews, 8, 18. doi:10.1186/s13643-019-0980-1 30808419 PMC6390353

[R28] Kimmes JG, Mallory AB, Spencer C, Beck AR, Cafferky B, & Stith SM (2019). A meta-analysis of risk markers for intimate partner violence in same-sex relationships. Trauma Violence and Abuse, 20(3), 374–384. doi:10.1177/1524838017708784 29333967

[R29] Liu H, Longshore D, Williams JK, Rivkin I, Loeb T, Warda US, … Wyatt G (2006). Substance abuse and medication adherence among HIV-positive women with histories of child sexual abuse. AIDS and Behavior, 10(3), 279–286. doi:10.1007/s10461-005-9041-y 16501869 PMC4398018

[R30] Lloyd S, & Operario D (2012). HIV risk among men who have sex with men who have experienced childhood sexual abuse: systematic review and meta-analysis. AIDS Education and Prevention, 24(3), 228–241. doi:10.1521/aeap.2012.24.3.228 22676462

[R31] *Markowitz SM, O’Cleirigh C, Hendriksen ES, Bullis JR, Stein M, & Safren SA (2011). Childhood sexual abuse and health risk behaviors in patients with HIV and a history of injection drug use. AIDS and Behavior, 15(7), 1554–1560. doi:10.1007/s10461-010-9857-y 21161362 PMC3638763

[R32] Mathews B, & Collin-Vézina D (2019). Child sexual abuse: Toward a conceptual model and definition. Trauma, Violence, & Abuse, 20(2), 131–148. 10.1177/1524838017738726 PMC6429628 29333990

[R33] McCall J, Lauridsen-Hoegh P, Unger D, Phillips JC, & Kille J (2018). Childhood sexual abuse in a population of patients living with HIV: Prevalence and impact. Journal of the Association of Nurses in AIDS Care, 29(3), 466–474. doi:10.1016/j.jana.2017.12.001 29306671

[R34] McGeough BL, & Sterzing PR (2018). A systematic review of family victimization experiences among sexual minority youth. Journal of Primary Prevention, 39(5), 491–528. doi:10.1007/s10935-018-0523-x PMC6408293 30206750

[R35] Meade CS, Hansen NB, Kochman A, & Sikkema KJ (2009). Utilization of medical treatments and adherence to antiretroviral therapy among HIV-positive adults with histories of childhood sexual abuse. AIDS Patient Care and STDS, 23(4), 259–266. doi:10.1089/apc.2008.0210 19260772 PMC2856435

[R36] Mugavero M, Ostermann J, Whetten K, Leserman J, Swartz M, Stangl D, & Thielman N (2006). Barriers to antiretroviral adherence: The importance of depression, abuse, and other traumatic events. AIDS Patient Care and STDS, 20(6), 418–428. doi:10.1089/apc.2006.20.418 16789855

[R37] Mugavero MJ, Pence BW, Whetten K, Leserman J, Swartz M, Stangl D, & Thielman NM (2007). Childhood abuse and initial presentation for HIV care: An opportunity for early intervention. AIDS Care, 19(9), 1083–1087. doi:10.1080/09540120701351896 18058391

[R38] O’Leary A, Purcell D, Remien RH, & Gomez C (2003). Childhood sexual abuse and sexual transmission risk behaviour among HIV-positive men who have sex with men. AIDS Care, 15(1), 17–26. doi:10.1080/0954012021000039725 12655830

[R39] Pantalone DW, Horvath KJ, Hart TA, Valentine SE, & Kaysen DL (2015). Traumatic revictimization of men who have sex with men living with HIV/AIDS. Journal of Interpersonal Violence, 30(9), 1459–1477. doi:10.1177/0886260514540802 24989040

[R40] *Pantalone DW, Valentine SE, Woodward EN, & O’Cleirigh C (2018). Syndemic indicators predict poor medication adherence and increased health care utilization for urban HIV-positive men who have sex with men. Journal of Gay and Lesbian Mental Health, 22(1), 71–87. doi: 10.1080/19359705.2017.1389794 30976378 PMC6456071

[R41] Paxton KC, Myers HF, Hall NM, & Javanbakht M (2004). Ethnicity, serostatus, and psychosocial differences in sexual risk behavior among HIV-seropositive and HIV-seronegative women. AIDS and Behavior, 8(4), 405–415. doi:10.1007/s10461-004-7325-2 15690114

[R42] Pence BW, Mugavero MJ, Carter TJ, Leserman J, Thielman NM, Raper JL, … Whetten K (2012). Childhood trauma and health outcomes in HIV-infected patients: an exploration of causal pathways. Journal of Acquired Immune Deficiency Syndromes, 59(4), 409–416. doi:10.1097/QAI.0b013e31824150bb 22107822 PMC3299853

[R43] Pretty C, O’Leary DD, Cairney J, & Wade TJ (2013). Adverse childhood experiences and the cardiovascular health of children: A cross-sectional study. BMC Pediatrics, 13, 208. doi:10.1186/1471-2431-13-208 24344611 PMC3878623

[R44] *Sauceda JA, Wiebe JS, & Simoni JM (2016). Childhood sexual abuse and depression in Latino men who have sex with men: Does resilience protect against nonadherence to antiretroviral therapy? Journal of Health Psychology, 21(6), 1096–1106. doi:10.1177/1359105314546341 25156387 PMC4476931

[R45] Sikkema KJ, Ranby KW, Meade CS, Hansen NB, Wilson PA, & Kochman A (2013). Reductings in traumatic stress following a coping intervention were mediated by decreases in avoidant coping for people living with HIV/AIDS and childhood sexual abuse. Journal of Consulting and Clinical Psychology, 81(2), 274–283. doi: 10.1037/a0030144. 23025248 PMC3615135

[R46] Sikkema KJ, Watt MH, Drabkin AS, Meade CS, Hansen NB, & Pence BW (2010). Mental health treatment to reduce HIV transmission risk behavior: A positive prevention model. AIDS and Behavior, 14(2), 252–262. doi:10.1007/s10461-009-9650-y 20013043 PMC2835810

[R47] Spies G, Afifi TO, Archibald SL, Fennema-Notestine C, Sareen J, & Seedat S (2012). Mental health outcomes in HIV and childhood maltreatment: a systematic review. Systematic Reviews, 1, 30. doi:10.1186/2046-4053-1-30 22742536 PMC3441909

[R48] Thornton CP, & Veenema TG (2015). Children seeking refuge: A review of the escalating humanitarian crisis of child sexual abuse and HIV/AIDS in Latin America. Journal of the Association of Nurses in AIDS Care, 26(4), 432–442. doi:10.1016/j.jana.2015.01.002 25769757

[R49] University of Oxford and Ottawa Health Research Institute (2015). PRISMA: Transparent Reporting of Systematic Reviews and Meta-Analyses. Retrieved from http://www.prisma-statement.org

[R50] *Watt MH, Dennis AC, Choi KW, Ciya N, Joska JA, Robertson C, & Sikkema K,J (2017). Impact of sexual trauma on HIV care engagement: Perspectives of female patients with trauma histories in Cape Town, South Africa. AIDS and Behavior, 21(11), 3209–3218. 27866288 10.1007/s10461-016-1617-1 PMC5438301

[R51] Whetten K, Leserman J, Lowe K, Stangl D, Thielman N, Swartz M, … Van Scoyoc L (2006). Prevalence of childhood sexual abuse and physical trauma in an HIV-positive sample from the deep south. American Journal of Public Health, 96(6), 1028–1030. doi:10.2105/ajph.2005.063263 16670226 PMC1470636

[R52] Whetten K, Reif S, Toth M, Jain E, Leserman J, & Pence BW (2012). Relationship between trauma and high-risk behavior among HIV-positive men who do not have sex with men (MDSM). AIDS Care, 24(11), 1453–1460. doi:10.1080/09540121.2012.712665 22909318 PMC3484220

[R53] *Whetten K, Shirey K, Pence BW, Yao J, Thielman N, Whetter R,…, & Reddy E CHAT Research Team. (2013). Trauma history and depression predict incomplete adherence to antiretroviral therapies in a low income country. PLoS One, 8(10):e74771. doi: 10.1371/journal.pone.0074771 24124455 PMC3790775

[R54] Whetten K, Whetten RA, Ostermann J, & Itemba D (2008). Trauma, anxiety and reported health among HIV-positive persons in Tanzania and the US Deep South. AIDS Care, 20(10), 1233–1241. doi:10.1080/09540120801918636 18608062

[R55] Willie TC, Overstreet NM, Sullivan TP, Sikkema KJ, & Hansen NB (2016). Barriers to HIV medication adherence: Examining distinct anxiety and depression symptoms among women living with HIV who experienced childhood sexual abuse. Behavioral Medicine, 42(2), 120–127. doi:10.1080/08964289.2015.1045823 26010763 PMC4710561

[R56] Wilson SM, Sikkema KJ, & Ranby KW (2014). Gender moderates the influence of psychosocial factors and drug use on HAART adherence in the context of HIV and childhood sexual abuse. AIDS Care, 26(8), 959–967. doi:10.1080/09540121.2013.873765 24410324 PMC4156823

[R57] Winzer L, Krahe B, & Guest P (2017). The scale of sexual aggression in Southeast Asia: A review. Trauma, Violence & Abuse, 20(5), 595–612. 10.1177/1524838017725312 29333964

[R58] *Young-Wolff KC, Sarovar V, Sterling SA, Leibowitz A, McCaw B, Hare CB,…Satre DD (2019) Adverse childhood experiences, mental health, substance use, and HIV-related outcomes among persons with HIV. AIDS Care, 31(10), 1241–1249. 30887831 10.1080/09540121.2019.1587372 PMC6675575

[R59] Zanarini MC, Yong L, Frankenburg FR, Hennen J, Reich DB, Marino MF, & Vujanovic AA (2002). Severity of reported childhood sexual abuse and its relationship to severity of borderline psychopathology and psychosocial impairment among borderline inpatients. Journal of Nervous and Mental Disease, 190(6), 381–387. doi:10.1097/00005053-200206000-00006 12080208

