# Supplementary material for: Childhood Sexual Trauma and Antiretroviral Therapy Adherence: A Mixed-Methods Systematic Review
Source: AIDS Behav. Author manuscript; Available in PMC 2022 Feb 1. (PMC7854942; doi:10.1007/s10461-020-03009-7)
Supplement: 10461_2020_3009_MOESM1_ESM [file NIHMS1623515-supplement-10461_2020_3009_MOESM1_ESM.docx]

**Appendix**

**PubMed Search Terms**

**Concept 1** – Childhood Sexual Trauma

MeSH

Adverse Childhood Experiences [MeSH]

Adult Survivors of Child Abuse [MeSH]

Battered Child Syndrome [MESH]

Child Abuse [MeSH]

Child Abuse, Sexual [MeSH]

Domestic Violence [MeSH]

Human Trafficking [MeSH]

Incest [MeSH]

Intimate Partner Violence [MeSH]

Psychological Trauma [MeSH]

Rape [MeSH]

Sex Offenses [MeSH]

Sex Workers [MeSH]

Trauma and Stressor Related Disorders [MeSH]

abuse [tw]

abused [tw]

abusive [tw]

adverse childhood experience* [tw]

adverse childhood event* [tw]

battered [tw]

coercion [tw]

dating violence [tw]

domestic violence [tw]

human trafficking[tw]

incest [tw]

IPV [tw]

intimate partner violence [tw]

maltreatment [tw]

prostitut* [tw]

rape* [tw]

sex worker* [tw]

sexual assault* [tw]

sexual violence [tw]

sexually assault* [tw]

torture* [tw

trauma* [tw]

victimization [tw]

**Concept 2 – Child**

Adolescent [MeSH]

Child[MeSH]

adolescen* [tw]

boys [tw]

child* [tw]

daughter* [tw]

girls [tw]

juvenile* [tw]

minor* [tw]

paediatric [tw]

pediatric [tw]

pupil*[tw]

school* [tw]

school-aged [tw]

son [tw]

sons [tw]

student* [tw]

teen* [tw]

youth [tw]

**Concept 3 – Antiretroviral**

MeSH

Acquired Immunodeficiency Syndrome [MeSH]

Anti-HIV Agents [MeSH]

Anti-Retroviral Agents [MeSH]

Antiretroviral Therapy, Highly Active [MeSH]

HIV [MeSH]

HIV Infections [MeSH]

acquired immunodeficiency syndrome [tw]

acquired immunedeficiency syndrome [tw]

acquired immune deficiency syndrome [tw]

acquired immuno deficiency syndrome [tw]

AIDS [tw]

antiretroviral* [tw]

anti-retroviral* [tw]

antiviral* [tw]

ART [tw]

ARTs [tw]

ARV [tw]

ARVs [tw]

HAART [tw]

HIV [tw]

HIV/AIDS [tw]

human immunedeficiency virus [tw]

human immunodeficiency virus [tw]

human immune deficiency virus [tw]

human immuno deficiency virus [tw]

PLWA [tw]

PLWH [tw]

PLWHA [tw]

**Concept 4: Adherence**

Medication Adherence [MeSH]

Patient Compliance [MeSH]

Treatment Adherence and Compliance [MeSH]

Treatment Refusal [MeSH]

adheren* [tw]

complian* [tw]

non-adheren* [tw]

non-complian* [tw]

nonadheren* [tw]

noncomplian* [tw]

refusal [tw]
